# A concept for human use of real-time and remote monitoring of diabetic subjects using intermittent scanned continuous glucose measurement

**DOI:** 10.1186/s12938-024-01217-z

**Published:** 2024-02-28

**Authors:** Jhon E. Goez-Mora, Natalia Arbeláez-Córdoba, Norman Balcazar-Morales, Pablo S. Rivadeneira

**Affiliations:** 1 Grupo GITA, Facultad de Minas, Universidad Nacional de Colombia, Carrera 80#65-223, 050001 Medellín, Colombia; 2 Grupo GENMOL, Departamento de Fisiología y Bioquímica, Facultad de Medicina, Universidad de Antioquia, 050001 Medellín, Colombia

**Keywords:** Diabetes mellitus, Interstitial glucose, Flash glucose monitoring system, Real-time and remote monitoring

## Abstract

**Background:**

Flash glucose monitoring systems like the FreeStyle Libre (FSL) sensor have gained popularity for monitoring glucose levels in people with diabetes mellitus. This sensor can be paired with an off-label converted real-time continuous glucose monitor (c-rtCGM) plus an ad hoc computer/smartphone interface for remote real-time monitoring of diabetic subjects, allowing for trend analysis and alarm generation.

**Objectives:**

This work evaluates the accuracy and agreement between the FSL sensor and the developed c-rtCGM system. As real-time monitoring is the main feature, the system's connectivity was assessed at 5-min intervals during the trials.

**Methods:**

One week of glucose data were collected from 16 type 1 diabetic rats using the FSL sensor and the c-rtCGM. Baseline blood samples were taken the first day before inducing type 1 diabetes with streptozotocin. Once confirmed diabetic rats, FSL and c-rtCGM, were implanted, and to improve data matching between the two monitoring devices, the c-rtCGM was calibrated to the FSL glucometer readings. A factorial design 2 × 3^3 and a second-order regression was used to find the base values of the linear model transformation of the raw data obtained from the sensor. Accuracy, agreement, and connectivity were assessed by median absolute relative difference (Median ARD), range averaging times, Parkes consensus error grid analysis (EGA), and Bland–Altman analysis with a non-parametric approach.

**Results:**

Compared to the FSL sensor, the c-rtCGM had an overall Median ARD of 6.58%, with 93.06% of results in zone A when calibration was not carried out. When calibration frequency changed from every 50 h to 1 h, the overall Median ARD improved from 6.68% to 2.41%, respectively. The connectivity evaluation showed that 95% of data was successfully received every 5 min by the computer interface.

**Conclusions and clinical importance:**

The results demonstrate the feasibility and reliability of real-time and remote subjects with diabetes monitoring using the developed c-rtCGM system. Performing calibrations relative to the FSL readings increases the accuracy of the data displayed at the interface.

## Introduction

The global problem of diabetes mellitus has led many researchers to focus on mitigating the effects of this disease, where monitoring and controlling blood sugar levels is a very relevant factor in avoiding macro- and microvascular complications in persons with diabetes.

Commercially available continuous glucose monitor (CGM) systems endorsed by the United States Food and Drug Administration have increased use in humans and animals. CGMs offer advantages over single glucose measurements by showing glucose evolution throughout the day, trends, and avoiding the constant inconvenience of obtaining blood samples [1–3].

Consequently, CGMs are satisfactory for diabetic subject care, reducing stress and suffering from blood sampling [4–6]. In particular, the intermittent scanned continuous glucose monitoring system, called FreeStyle Libre sensor (FSL), developed by Abbott Diabetes Care has recently gained popularity [7, 8]. The readings obtained by this sensor have been validated in different investigations with humans, and several animals as cats, dogs, and rodents. These show maximum deviations of 15% from blood reference values, and readings remain in zones A and B of the Parkes consensus error grid analysis (EGA). As a result, FSL readings are acceptable for monitoring diabetic subjects despite known latency concerning blood samples [6–15].

Although FSL sensors show potential to improve the quality of life of diabetic subjects, it remains challenging for many persons to do the correct monitoring, mainly while the person who is taking care is not physically with the diabetic subject. To overcome this, off-label transmitters have been developed to be paired with the FSL sensor and to send glucose measurements in real time to an electronic device such as a computer or smartphone, which can share data with remote devices. Therefore, received data can be displayed in applications in local and/or remote interfaces to plot values, compute trends, and generate alarms. The MiaoMiao transmitter (MMT) is available commercially allowing data transmission from FSL sensors via Bluetooth to computer devices using commercial (or open access) apps such as Tomato, xDrip+, Spike, and Glimp, among others [16]. This setup and its features plus its low cost compared with other solutions could be also very appealing for owners of diabetic pets [2, 3].

These apps are software platforms designed for managing data, plotting data and trends, generating alarms, and controlling insulin dosage through a pump connected to the system. However, lacking FSL’s official calibration algorithm can lead to significant differences from the FSL sensor readings. Many of these apps are open source, but their calibration methods are generally protected and not shared in the developing community.

Previous work [16] showed that the overall mean absolute relative difference (MARD) between the FSL sensor and the measurements plotted by these apps (for example, Xdrip+) is around 15%, which is the maximum limit accepted by the glucose measurement standard and has a notorious dependence on the number of calibrations.

In this work, an ad hoc computer/smartphone interface is developed to support real-time monitoring of diabetic subjects using the MMT, including a calibration method to match FSL data. The tool was tested on 16 type 1 diabetic male rats of the Wistar strain using an FSL sensor for 1 week. The MMT wirelessly sent raw measurements to the ad hoc app, where a calibration method matches RAW data, and the resulting value is displayed in the interface. After completing the trial, the accuracy, agreement, and connectivity of both FSL and converted real-time CGM (c-rtCGM) systems were assessed using Bland–Altman plots, Median ARD, and Parkes consensus EGA. Although this was tested using animal models, it is intended as a concept for human use.

## Materials and methods

### Devices

The FSL sensor (version 1) is an intermittently scanned, factory-calibrated glucose monitor requiring a 1-h warmup and 14-day wear lifetime. Its dimensions are 35 mm × 5 mm and weighs 5 g; it also has a water resistance of IP27. Installation requires penetrating the skin with a 1-cm G22 needle to insert a filament into interstitial space from where it takes readings equivalent to plasma glucose or interstitial glucose (IG) concentration. Raw data are retrieved through near field communication (NFC, where the reading distance is under 4 cm) protocol to readers such as FSL official readers, smartphones (with an official app), or off-label transmitters, which transform raw data to the actual measurement. The sensor can measure at each minute, but the reading is only performed when scanned with a reader. The sensor has a memory capacity to record up to the last 8 h of data but with a sampling of 15 min.

MiaoMiao 2 is a transmitter paired with the FSL to obtain a c-rtCGM system using NFC to read raw data every 5 min automatically. It sends the data via Bluetooth to a paired computer/smartphone interface without going through the official FSL conversion algorithm. It has a 2-week rechargeable battery and IP67 protection. Its dimensions are 35 mm × 20 mm x 10 mm, weighing 6 g.

A laptop with a 64-bit Windows 10 operating system, 64 GB of RAM, Intel processor Intel(R) Xeon(R) W-10855 M CPU @ 2.80 GHz 2.81 GHz, and Bluetooth Qualcomm QCA61 × 4A was used to process and monitor displayed data.

Remark: No device manufacturers were involved in the present study.

### Animals

A total of 16 male rats of the Wistar strain, SPF microbiological condition, 14–16 weeks old, weighing 400–600 g, were considered in this study. They were housed individually in polycarbonate boxes measuring 45 cm × 25 cm × 20 cm with a 5 cm layer of previously sterilized chips to avoid removal of the devices. The macro-environment conditions were temperature controlled at 21 °C ± 2 °C, 50–65% humidity with 16–20 air changes/hour, and artificial lighting with white light on a light/dark 12/12-h cycle regulated by a timer. Food for laboratory rodents, brand 5010—Laboratory Autoclavable Rodent Diet (LabDiet®), was supplied with a minimum of 23% crude protein, 4.5% crude fat, and a maximum of 6% fiber ad libitum and water previously sterilized.

For the induction of type I diabetes, rats were fasted for 6 h and subsequently administered streptozotocin at 60 mg/kg intraperitoneally. Afterward, a 10% sucrose solution was provided in the drinker for 24 h before returning to regular water.

After 24 h of diabetes induction, general clinical and blood glucose monitoring began using the FSL reader, which also works as a glucometer. A drop of blood was taken by puncture of the saphenous vein every 4 h. Diabetes was confirmed, and insulin dosing started when glucose exceeded 150 mg/dL. Daily clinical evaluations ensured animal welfare according to the guidelines for the care and use of laboratory animals [17].

Rapid-acting insulin NovolinR® for human use (rDNA) and long-acting recombinant insulin from Lantus® were administered to reproduce the standard subcutaneous basal-bolus treatment during the disease.

### Sensor and transmitter installation

**Fig. 1:**
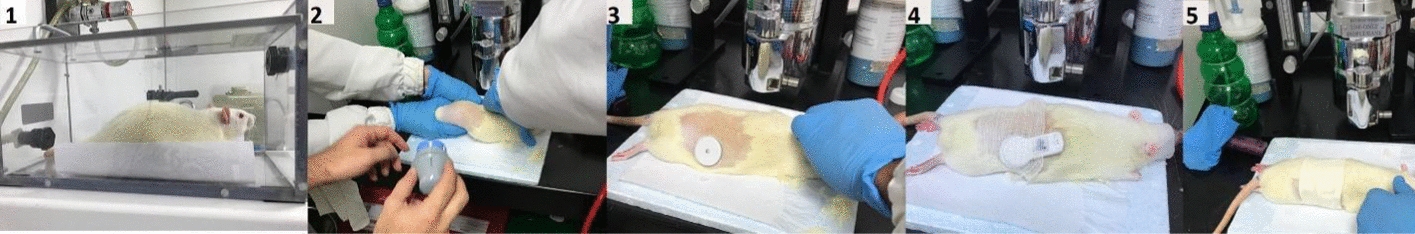
The sensor and c-rtCGM system are installed in rats after induction with type I diabetes. (1) Induction in an inhalation anesthesia chamber with isoflurane. (2) Hair removal of the dorsal and ventral areas of the rats under anesthesia and maintenance of this inhalation route with a facial mask. (3) Installation of the sensor and verification of its insertion perpendicularly. (4) Installation of the transmitter on top of the sensor, adhesion to the skin and fixation with gauze. (5) Covering the sensor and transmitter with sticking plaster

After type 1 diabetes induction, FSL sensors and c-rtCGM were installed in each anesthetized rat (isoflurane induction 5%, maintenance 1–2%) to facilitate and reduce manipulation stress since it is a painless procedure. The sensor was introduced subcutaneously, the transmitter was attached, and the system was fixed with gauze and covered with a drape. Figure 1 shows the installation and fixing procedure. The sensor was activated using the FSL reader, and the transmitter was paired via Bluetooth to the computer interface.

The rats were placed again in the corresponding box until recovery the presence of abnormal signs or immediate adverse reactions after the procedure was monitored. The implantation of the c-rtCGM system and the FSL sensor did not restrict mobility. Once the sensor was activated, the monitoring system started to receive and show data in the computer interface. In addition, three blood glucose measurements per day were obtained to verify that sensor values were within the normal accuracy range between 100 and 180 mg/dL ± 20 mg/dL.

### Monitoring interface

**Fig. 2:**
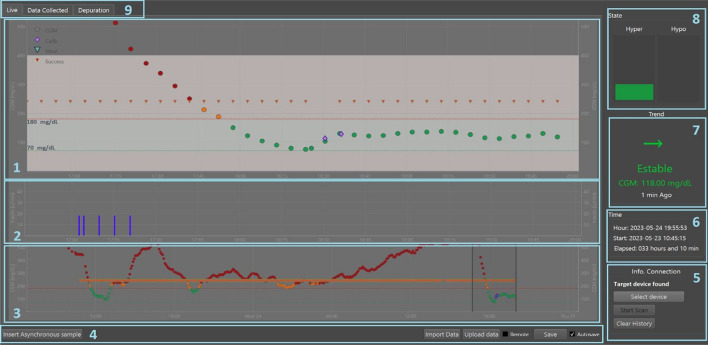
Continuous glucose monitoring interface. (1) Sensor glycemic recording (CGM), glucometer asynchronous display (Calib), carbohydrates (Meal), and successful glycemic recording (Success). (2) Insulin injected. (3) Moving slider for time window in 1 and 2. (4) Data options. (5) Transmitter connection information. (6) Record of elapsed time. (7) Current value and trend of glycemia. (8) Hyper and hypoglycemia alarms. (9) Real-time registration, collected data, and system logs

The monitoring interface implements functions to match, record, and display blood glucose levels from the sensor, record glucose from blood samples, and inject insulin and carbohydrate intake. Figure 2 shows, in general terms, the information visualized in the interface that supports the owners' decision-making. The central panel (1) plots blood glucose measurements in color-coded circles that change according to their range of values: green from 70 to 180 mg/dL, yellow between 50 and 70 mg/dL, and 180–220 mg/dL and red for values less than 50 and greater than 220 mg/dL. Orange triangles mark successful 5-min data receipt, and purple diamonds record asynchronous measurements with glucometer or FSL sensor readings. Green triangles record consumed carbohydrates.

Panel (2) shows the insulin injected through subcutaneous injections or a connected insulin pump with the interface. In Panel (3), there is a slider with which the user can modify the time window to be visualized in Panels 1 and 2. Panels (4) and (9) provide options to add asynchronous samples, save, import, or upload data. Panel (5) shows Bluetooth connection status with the transmitter. Panel (6) displays the total interface execution time. Panels (7) and (8) show trends and alarms for the current blood glucose concentration of the subject with color changes according to the glucose range.

The interface binds to the selected transmitter and collects data. If the connection is made every 5 min, it is marked as successful with an orange triangle. If the transmitter is out of range, it requests reconnection every minute until successful, retrieving missing data every 15 min for up to 8 h that the sensor stores. The interface also registers calibration points with purple diamonds and carbohydrate consumption (meals) with green triangles (however, in this study, meals were not registered). The data collection option in Panel 9 records the animal's date, time, raw sensor value, and encoded glycemia value. The depuration option opens a window that logs interface commands. Data can be exported or imported in CSV formats.

The monitoring interface obtains the sensor's primary data (raw) and then applies a mathematical transformation to record the current blood glucose measurement. This transformation is a linear equation where its intercept is updated at each calibration using the measurements of the asynchronous samples obtained by the glucometer or the FSL measurement to adjust the values of the contraction “a” and vertical translation “b” of Eq. 1 to minimize the error:

1 $$G=\left[\frac{CGM\_RAW}{a}\right]-b.$$

Equation 1: A linear model to transform raw data to actual glucose concentration measurement, where G means glucose concentration (mg/dL).

### Study design

The study compares the glucose readings from the commercial FSL sensor and the combination of the MMT and an ad hoc computer/smartphone interface as a tool for monitoring diabetic subjects.

Sixteen male Wistar rats were included in the trial. First, the sensors and transmitter were installed in healthy rats for baseline data. Then, diabetes was induced by injecting streptozotocin. Once declared diabetic (blood glucose >  = 150 mg/dL), standard basal-bolus treatment began. The interface was monitored every 5 min around the clock, while FSL readings were taken manually for up to 10 h daily due to staffing, as the sensor requires the laboratory staff to physically take the reader and bring it closer to the animal. The maximum duration of the experiment was 1 week per subject. Blood glucose sampling verified the sensor and FSL readers.

### Data analysis

In the first stage, a factorial design 2 × 3^3 with two replicates is used to find the base values of Eq. 1. The first factor is qualitative with two levels: sensor location (dorsal, ventral), the second factor is qualitative with three levels: blood glucose range (Hypoglycemic < 80 mg/dL, 80 mg/dL < normoglycemic < 180 mg/dL, Hyperglycemic > 180 mg/dL), the third factor is quantitative with three levels: contraction of a linear transformation (6.8, 7.3, 8.6), the fourth factor is quantitative with three levels: vertical translation of linear transformation (15, 20, 30). The initial values of the contraction and vertical translation were taken from tests before the experiment. The variable response is the average of the difference between the values of the FSL and the c-rtCGM.

The objective is to find the best combination between the base values of the linear transformation and the sensor location considering the blood glucose ranges. The analysis of variance (ANOVA) is used to determine the best combination of the factors and the response variable of a second-order regression is optimized to obtain the linear transformation values that are used as a basis for analyses where the interface is not calibrated during data collection.

A linear transformation is applied to the RAW values obtained by the c-rtCGM system to record glucose, which is then compared with the FSL reference data. After the tests, calibrations are performed with the reference data every 200, 100, 64, 32, 16, and 4 samples, equivalent to a period between calibrations of 50, 25, 16, 8, 4, and 1 h, respectively.

For each data set with and without calibration, pairs are matched every 15 min, corresponding to the data frequency of the FSL system. Accuracy metrics are calculated on these data pairs, including Media ARD, average, standard deviation (SD), and coefficients of variation (CV) of the overall data and percentage of time in glycemic ranges (IG < 70, 70 $\le$ IG $\le$ 180, 180 $\le$ IG $\le$ 250, 250 $\le$ IG $\le$ 300, IG $\ge$ 300 mg/dL). The Parkes consensus EGA is also applied, where zones A and B represent acceptable medical precision in the measurement between the c-rtCGM system and the FSL sensor. Finally, the Bland–Altman plot is visually analyzed for agreement between the two measurements to estimate differential and proportional biases.

## Results

Throughout the experiment, 15,490 usable data points were collected, accounting for the 16 rats, of which 95.2% had successful 5-min MiaoMiao to c-rtCGM Bluetooth transmission. Missed data were recovered from the 8-h sensor backup upon reconnecting every 15 min. Only two cases were recorded to exceed the 8 h of sensor storage before reestablishing connection, losing around 5 h of data in both cases, corresponding to only 0.77% of the total data.

For the design of the experiment, 108 experimental runs were made with fully randomized replications. The ANOVA is presented in Table 1, all main effects are significant and for the interactions only A*B, A*C, and B*C are statistically significant. The coefficient of determination *R*^2 = 95.17% and the *R*^2 adjusted = 93.69% confirm that the chosen factors explain the model's behavior.

**Table 1:** Analysis of variance

Source	DF	Seq SS	Contribution (%)	Adj SS	Adj MS	*F*-value	*P*-value
Model	25	93,753.1	95.17	93,753.1	3750.1	64.57	0
Linear	7	69,973.8	71.03	69,973.8	9996.3	172.11	0
A	1	13,218	13.42	13,218	13,218	227.58	0
B	2	15,579.7	15.81	15,579.7	7789.9	134.12	0
C	2	37,326.2	37.89	37,326.2	18,663.1	321.33	0
D	2	3849.9	3.91	3849.9	1925	33.14	0
2-way interactions	18	23,779.3	24.14	23,779.3	1321.1	22.75	0
A*B	2	7261.1	7.37	7261.1	3630.5	62.51	0
A*C	2	923.6	0.94	923.6	461.8	7.95	0.001
A*D	2	47.8	0.05	47.8	23.9	0.41	0.664
B*C	4	15,449.1	15.68	15,449.1	3862.3	66.5	0
B*D	4	20.6	0.02	20.6	5.1	0.09	0.986
C*D	4	77.1	0.08	77.1	19.3	0.33	0.856
Error	82	4762.5	4.83	4762.5	58.1		
Total	107	98,515.7	100.00				

Values reported in Software. © 2023 Minitab LLC. V 21, abbreviations: DF: degrees of freedom; Seq SS: sequential sum of squares; Adj SS: adjusted sum of squares; Adj MS: adjusted mean square, A: sensor location; B: blood glucose range; C: contraction; D: vertical translation

**Fig. 3:**
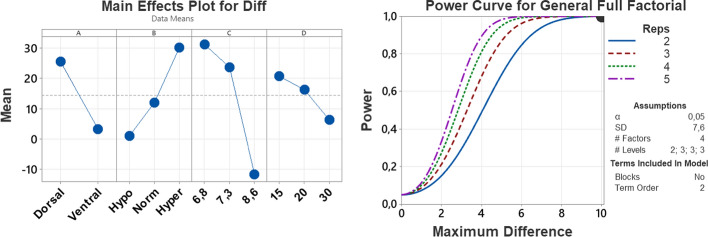
Main effects and power curve in Software. © 2023 Minitab LLC. Left panel: main effects plot for the difference between the FSL and the c-rtCGM system. Right panel: power curve, Diff: difference between the FSL and the c-rtCGM system; Reps: number of replicates; SD: standard deviation

Figure 3 shows the main effects plot, the location of the sensor in the ventral zone reduces the difference and the increase in blood glucose values increases the difference between the measurements regardless of the other factors. By analyzing the interactions between factors, a performance improvement can be determined when the value in the contraction of the linear transformation is 8.6. However, it is notable that it is possible to find a point between 7.3 and 8.6 that could have better results and for vertical translation values between 20 and 30 improve the precision of the c-rtCGM system. It is also verified that by using a maximum difference of 10 mg/dL and an SD of 7.6 mg/dL with two replicates, a statistical power of one is obtained.

**Fig. 4:**
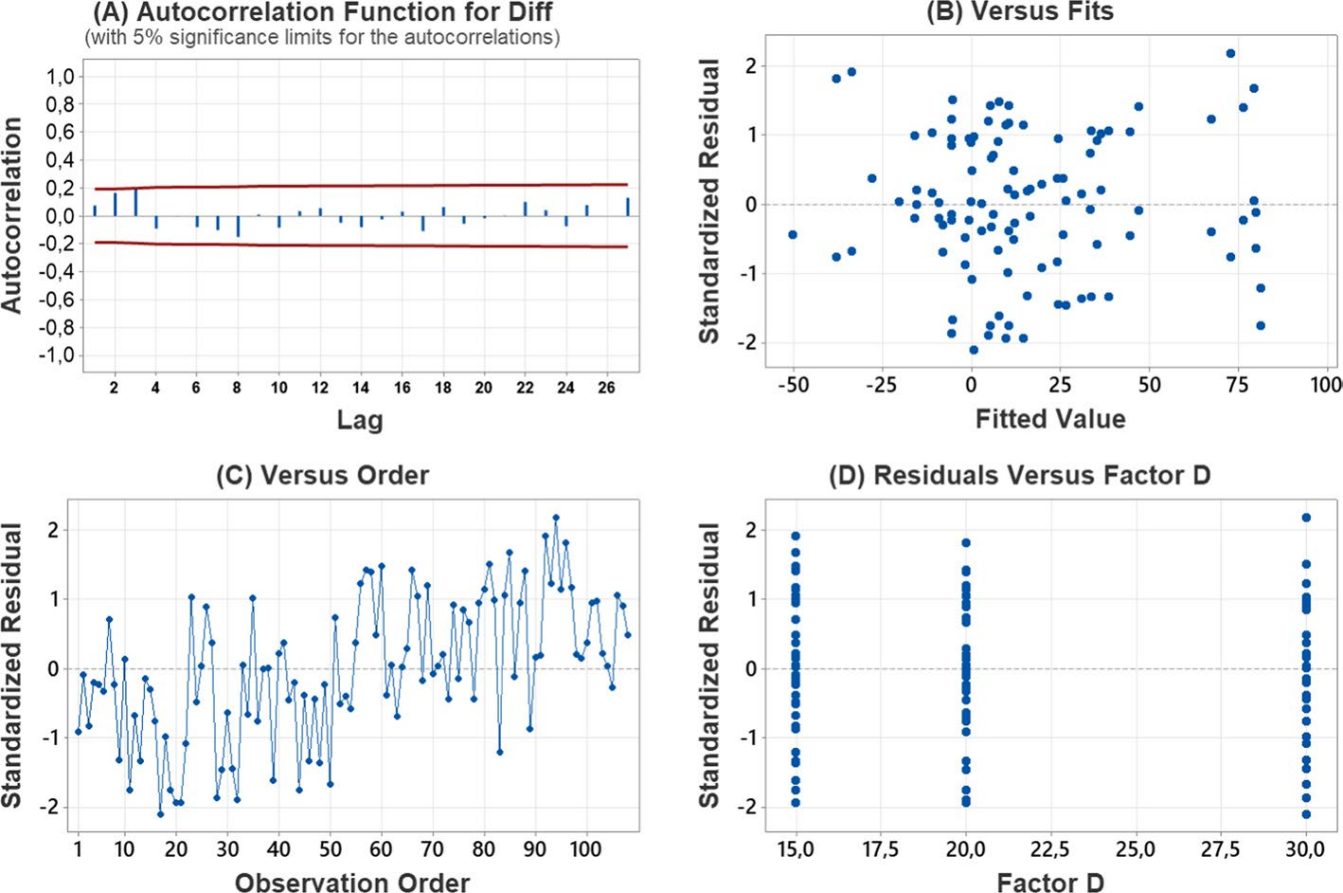
Validation of assumptions in Software. © 2023 Minitab LLC.** A** autocorrelation.** B** residuals versus fits.** C** residuals versus order.** D** residuals versus factor D, *N*: number of non-missing observations; SD: standard deviation; Diff: difference between the c-rtCGM system and FSL

For the validation of assumptions, the Kolmogorov–Smirnov test shows with a value of 0.082 and a *P*-value of 0.073 it is not possible to reject the null hypothesis of a normal distribution with the data collected from six rats. With Fig. 4 and the Durbin–Watson Statistic of 1.54891, it shows that there is no clear autocorrelation in the model. In the adjusted values versus the residuals, there is no bugle pattern or significant increase for any of the extremes, complying with the assumption of a constant variable or homoscedasticity. For the assumption of constant variance, it is observed in the points of the D factor level that the amplitude in the dispersion of the points is similar and for the independence in the graph of the residuals versus the order of the runs there is no explicit pattern.

Subsequently, a second-order regression model is proposed where the interactions CD, CA, DA, and DB are not statistically significant and the equations setting the ventral installation zone for each blood glucose range are: $difference=76.6-7.04C+0.09D-0.157CD$ for hypoglycemic, $difference=143.9-15.62C+0.07D-0.157CD$ for normoglycemic and $difference=357.9-43.32C+0.2D-0.157CD$ for hyperglycemic, reporting an ${R}^{2}=93.28\%$ and the ${R}^{2}adjusted=92.27\%$. The same verification of the assumptions of the regression model is carried out and optimization of the response is made with the objective of a zero average in the differences between the two measurement systems by fixing the location of the sensor in the ventral zone for each blood glucose range.

The results show that using a value of 7.7 in the contraction and a value close to 20 in the vertical translation of the linear transformation of Eq. 1 improves the difference between the FSL and the c-rtCGM system over all the ranges of blood glucose. These values are the ones used during the rest of the tests when calibrations are not performed with the asynchronous samples on the interface.

Of the total data, 4600 measurements were matched between the c-rtCGM system and the FSL sensor in the corresponding hours after cleaning glycemic values between 50 and 400 mg/dL across the 16 rats. The resulting average values, SD, and CV of the general sample and the percentages of time by ranges for the control data from FSL, along with the interface collected data with calibrations at set intervals and without calibrations, are reported in Table 2.

**Table 2:** Average glycemic values overall and by range

Data interval/lapse time (h)	FSL	None	200/50	100/25	64/16	32/8	16/4	4/1
Mean IG (mg/dL)	179.86 ± 63.06	190.90 ± 72.57	172.05 ± 58.70	173,22 ± 59.04	178.67 ± 64.67	177.12 ± 62.80	177.95 ± 61.70	180.25 ± 63.61
SD of IG (mg/dL)	67.61 ± 38.12	71.60 ± 39.43	66.41 ± 36.75	69.42 ± 38.24	70.54 ± 40.32	70.04 ± 39.62	70.78 ± 39.27	70,55 ± 39,00
CV (%)	36,77 ± 17,68	36,96 ± 17.39	38,19 ± 17,81	39,40 ± 18,40	38,76 ± 17,76	38,78 ± 18,11	39,06 ± 18,17	38,45 ± 17,92
Percentage of time in range (%)								
IG < 54 mg/dL	0 ± 0.43	0 ± 0.41	0 ± 1.29	0 ± 0.92	0 ± 1.10	0 ± 1.61	0 ± 1.10	0 ± 0.82
IG 54–70 mg/dL	3.08 ± 7.71	1.44 ± 6.10	4.93 ± 12.12	3.83 ± 16.13	4.32 ± 10.07	4.32 ± 12.49	4.32 ± 13.62	6.71 ± 7.81
IG 70–180 mg/dL	56.00 ± 31.33	55.35 ± 30.83	55.52 ± 29.83	54.31 ± 29.53	55.49 ± 29.77	54.55 ± 30.17	53.66 ± 30.46	53.51 ± 30.72
IG 180–250 mg/dL	39.47 ± 31.12	41.57 ± 31.31	37.07 ± 29.78	37.67 ± 29.46	38.01 ± 29.88	38.22 ± 29.81	38.78 ± 29.88	38.93 ± 30.50
IG 250–300 mg/dL	30.64 ± 29.80	32.34 ± 29.77	19.29 ± 49.22	20.61 ± 38.13	20.17 ± 48.51	24.12 ± 47.82	29.58 ± 29.58	30.80 ± 29.99
IG > 300 mg/dL	7.97 ± 22.61	15.35 ± 32.24	7.24 ± 27.05	11.59 ± 22.30	7.97 ± 25.28	7.97 ± 25.23	7.97 ± 24.61	7.97 ± 25.48

Values are reported as means ± SD, IG: interstitial glycemia concentration; FSL: control data; None: uncalibrated data; SD: standard deviation; h: hours

Table 3 reports the overall and range-specific Median ARD values for uncalibrated data and data calibrated by each interval. Overall, Median ARD was 6.58% without calibrations and 2.41% with hourly calibrations. The highest difference occurred in the IG $\le$ 70 mg/dL range, with 9.38% uncalibrated Median ARD and 5.83% with calibration.

**Table 3:** General and range-specific Median ARD between the c-rtCGM and the FSL systems

Data interval/lapse time (h)	None	200/50	100/25	64/16	32/8	16/4	4/1
Total Median ARD (%)	6.58	6.68	5.91	6.15	5.71	4.78	2.41
Median ARD (%) IG < 70 mg/dL	9.38	8.73	9.33	13.58	10.32	9.19	5.83
Median ARD (%) IG 70–180 mg/dL	6.73	7.28	6.37	6.01	5.87	5.06	2.58
Median ARD (%) IG 180–250 mg/dL	6.25	8.14	7.87	8.64	8.34	5.11	2.61
Median ARD (%) IG 250–300 mg/dL	8.30	5.05	5.17	5.34	4.16	3.50	1.92
Median ARD (%) IG > 300 mg/dL	4.83	4.25	3.66	6.33	4.06	3.23	1.60

Values are reported as percentages, Median ARD: median absolute relative difference; IG: interstitial glycemia concentration; None: uncalibrated data; h: hours

**Fig. 5:**
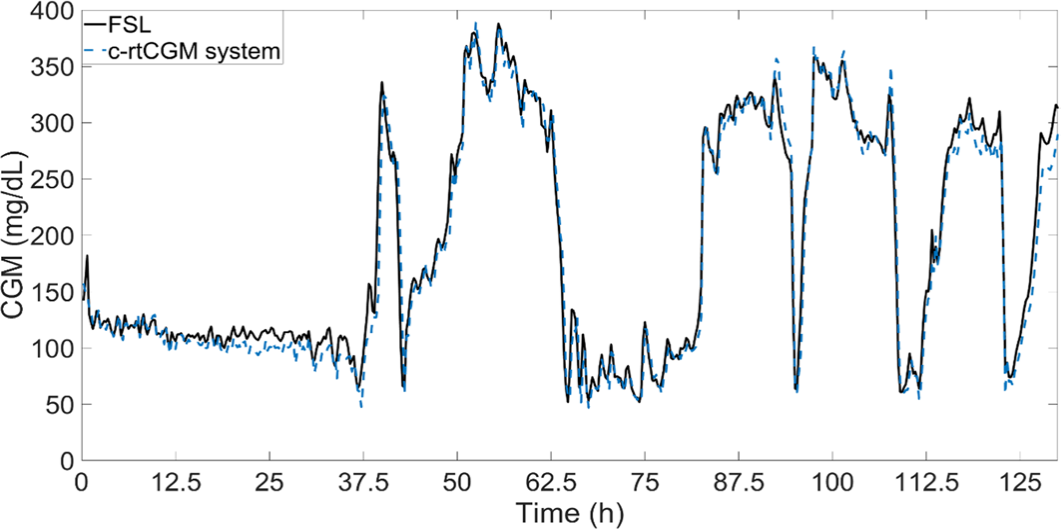
Time trace of the reported CGM for both devices in one rat for five days

Additionally, Fig. 5 shows the time trace with the values reported by the FSL and the c-rtCGM system in one of the subjects presenting average values of the differences between the devices by ranges: in hypoglycemia − 0.24 ± 9,210 mg/dL, in normoglycemia − 6,81 ± 14,08 mg/dL and in hyperglycemia − 5,53 ± 17,50 mg/dL.

**Fig. 6:**
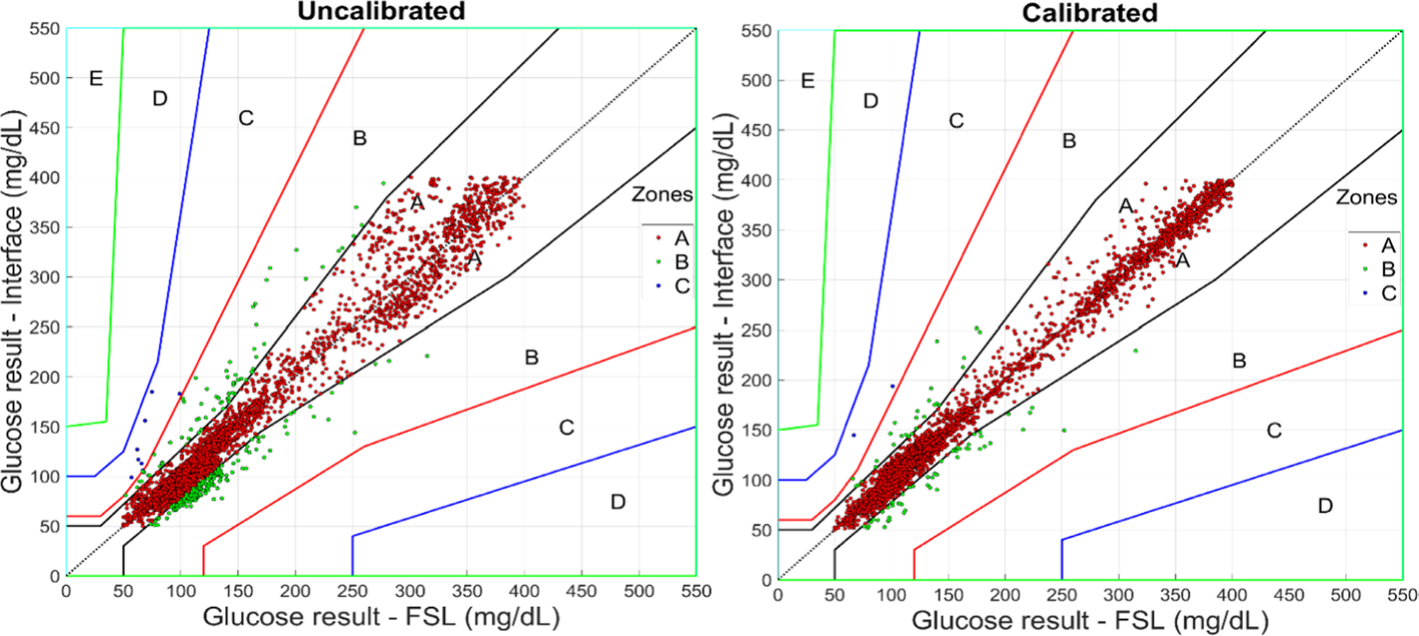
Parkes consensus error grid analysis. Left panel: results taken as reference the FSL sensor and the c-rtCGM system without performing calibrations. Right panel: results in the c-rtCGM system after calibrating every four samples collected at 15-min intervals

When considering the requirements of the EGA, data obtained with and without calibrations fell in zones A, B, and C. In Fig. 6, the left panel shows more excellent dispersion between the zones when no calibrations are made. The right panel shows the data from the most frequent calibration intervals (every four samples with intervals of 15 min), identifying that the distribution of the outer zones is concentrated and closer to the *x* = *y* axis of zone A. Table 4 reports the data by calibration intervals, showing the progressive shift of the data from the outer to the inner zones as the number of calibrations performed increases.

**Table 4:** Parkes consensus error grid analysis interval zones

Data interval/lapse time (h)	None	200/50	100/25	64/16	32/8	16/4	4/1
Zone A (%)	93.06	96.02	96.62	95.77	96.46	97.54	98.15
Zone B (%)	6.76	3.83	3.19	4.03	3.41	2.31	1.68
Zone C (%)	0.18	0.15	0.19	0.19	0.13	0.15	0.04
Zone D (%)	0.00	0.00	0.00	0.00	0.00	0.00	0.00
Zone E (%)	0.00	0.00	0.00	0.00	0.00	0.00	0.00

Values are reported as a percentage, h: hours

**Fig. 7:**
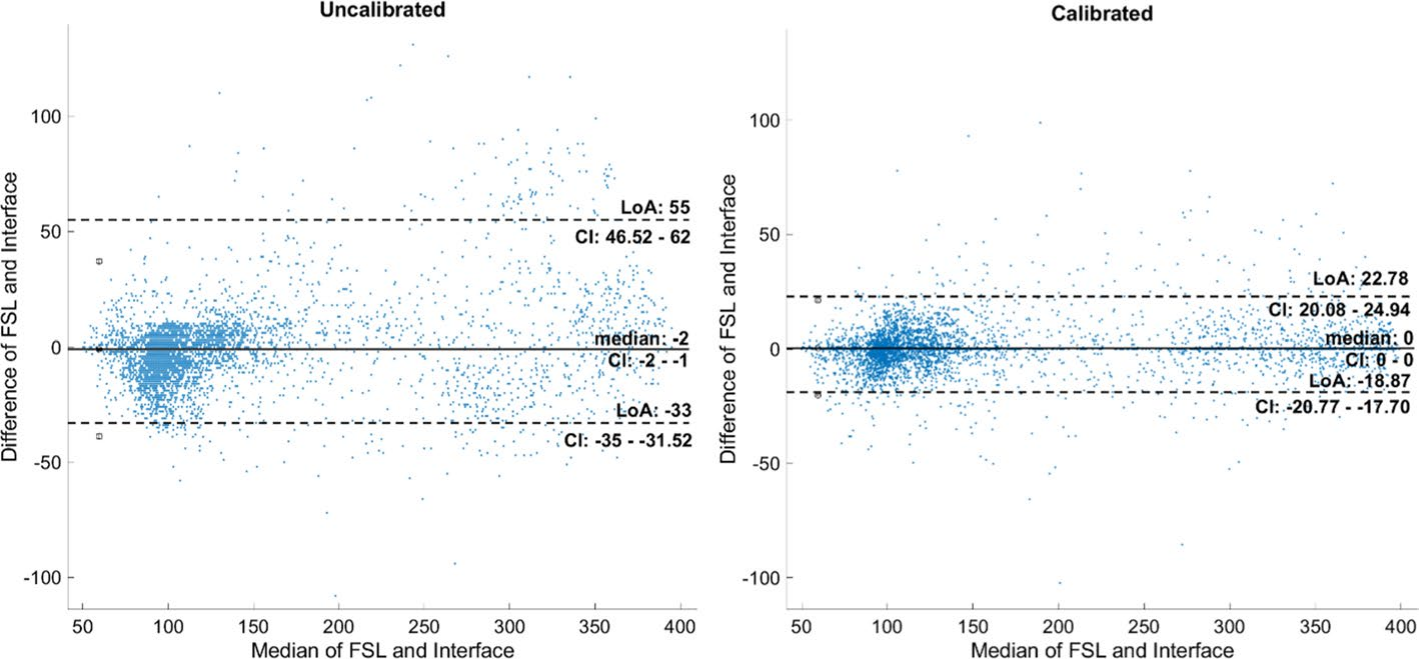
Bland–Altman graph. Left panel: results without performing calibrations. Right panel: results after calibrating every four samples collected at 15-min intervals

Figure 7 shows agreement improvement after performing the highest frequency of calibrations. The median difference shifted from -2 (95% confidence interval (CI) -2 to -1) to 0 (CI 0 to 0). In addition, the limits of agreement (LoA) were obtained from a non-parametric percentile of 2.5% and 97.5% [18, 19]. For data without calibration, LoA was − 33 to 55. For the system with calibrations, LoA were − 18.97 and 22.79, and all CI estimations were calculated with a bootstrap approach using 2000 samples [20]. Kolmogorov–Smirnov test of all data collected resulted in (*h* = 1, *p*-value = 7.0317e−86) and (*h* = 1, *p*-value = 2.0433e−94) for uncalibrated and calibrated data, respectively, indicating that neither case had a normal distribution of the differences. This is the reason for using the non-parametric Evaluation of Agreement.

## Discussion

An alternative surgical procedure to fix the sensor to the body of rodents with stitches [12] (which also requires sedating the animal and making an incision) can improve the success rate in installing the instruments, especially when looking for long-term monitoring of animals in preclinical trials.

For this reason, in this work, dorsal and ventral sensor placement was tried to identify a place that allows adequate blood irrigation for data collection. In the dorsal area, several problems with the sensor activation were found because this area has a considerable amount of adipose tissue, and therefore, there was not a good blood supply to the sensor filament, leaving the sensor unusable. In this area, the success rate of installation was only 25%. When the ventral area was considered, the success rate increased to 82% and the average difference between FSL and c-CGM decreased. Despite this improvement, there were still problems in installing the sensor due to the animal's size and having little space available that would allow adhesion to the skin and allow the animal to move freely. This problem may also occur in cats and dogs, but due to their size, the success rate in installation is expected to be higher than in rodents. During the trials, there were also cases in which the subjects did not complete the 2-week cycle because they removed the sensor, and despite trying to reinstall a new sensor, it did not activate correctly. This latter could be improved with pet training before device installation.

After data processing, the average subject recording was five days. Data points with IG > 400 mL/dL were cleaned, leaving 15,490 points from the 23,040 values collected. After pairing the remaining data with the FSL reader (which records values every 15 min), considering an average of 10 h of daily readings per animal, approximately 4600 data pairs were finally usable.

Of the total recorded data (23,040 values), 4.8% were recovered from the sensor memory, and the rest was received successfully every 5 min. Loss of connectivity occurred primarily because of the movement of the animals to another room to perform weighing procedures and blood samples, among others. Only one event caused the system to collapse overnight. The connection was reestablished the next day.

The comparison of the average values and specific glucose ranges between the FSL sensor and the c-rtCGM system in Table 2 shows a progressive improvement in the Median ARD and the other metrics when the number of calibrations increases. However, the CV, when considering calibrations, tends to increase the difference of the data with their arithmetic mean. This latter indicates that although the difference in average values decreases with calibrations, the linear transformation is insufficient to smooth and bring the following sample values closer to the reference data.

It is also observed that the percentage of glycemia by range begins to move from the highest to the lowest ranges as the number of calibrations increases, evidencing the tendency of the transformation to reduce the calculation of glycemia from the raw data while maintaining the general average close to the reference, but decreasing the average time in high blood glucose values. However, frequent calibration requires constantly manipulating the animal or getting closer to them, precisely what is sought to be reduced with continuous monitoring in real-time. Therefore, a tradeoff between the system's accuracy and the comfort of the diabetic subjects and people taking care of diabetic subjects must be established.

According to the EGA, 99.82% of the data are in zones A and B despite not performing calibrations. The increase in calibrations improves precision by concentrating the data on the *x* = *y* line, as seen in Fig. 6, and Table 4 shows the progressive transfer of the data located in zones C and B to zone A, managing to go from a concentration of 93.06% in zone A without calibrating to 98.15% with calibrations within 1 h. The calibrations help improve the correspondence between the control and interface data. However, the system itself, without calibrations, can concentrate the data so that according to this analysis, it complies with ISO 15197:2013, which indicates that 99% of the data must be contained in zones A and B to be considered an accurate device. In this case, what is wanted to demonstrate is that remote monitoring can be equivalent and reliable as the FSL sensor readings, of which its precision and validation as a monitoring system in animals have already been demonstrated [6, 15].

On the other hand, the visual analysis of the agreement in Fig. 7 allows us to identify that the biases due to the average difference in the uncalibrated measurements are negative and 2 times larger than those presented when calibrations are carried out every four samples. In addition, an approximately constant systematic error can be observed in both cases. The calibrations, apart from approximating the bias to zero, delimit the confidence ranges between which 95% of the differences between the FSL sensor and the c-rtCGM system are expected to be found. The results obtained in this work can be compared with those of [16], where they use Xdrip+ as the monitoring interface, demonstrating that using the RAW data despite not having calibrations improves the agreement of the differences and prevents values < 150 mg/dL the differences are negative and with higher values the difference is positive.

In conclusion, in this work, an extension of previous works related to monitoring diabetic subjects is carried out by comparing the precision and agreement of the FSL sensor and the c-rtCGM system. Using the factorial design and optimizing the response variable of the regression, it was possible to determine the base values of the linear transformation that present better performance when there are no calibrations of the asynchronous samples taken from the FSL. The results show a high agreement between the measurements of the two systems. This latter improves as the frequency of calibrations carried out by the user increases. However, it is also observed that by not performing calibrations, the differences between the two devices remain within an acceptable range in which can be stated that monitoring can be carried out remotely and is within the same range of precision as the FSL sensor, unlike applications such as Xdrip+, which by not performing calibration, the performance and agreement of the data decreases considerably. Reliable real-time and remote monitoring allows subjects with diabetes to have the benefits of using an FSL sensor. However, it provides the bonus of having a complete history, trends, alarms, and the possibility of intervention if an infusion pump is connected to the system without the need to be in contact with the subject with diabetes, nor that the measurements depend on this. Future work is necessary to find a way to improve the comfort of animals or humans when using this type of sensor in the long term.

## Data Availability

Not applicable.
